# Integrated behavioral health implementation and chronic disease management inequities: an exploratory study of statewide data

**DOI:** 10.1186/s12875-024-02483-5

**Published:** 2024-08-14

**Authors:** Gretchen J. R. Buchanan, Jerica M. Berge, Timothy F. Piehler

**Affiliations:** 1 Redleaf Center for Family Healing, Hennepin Healthcare Research Institute, 701 Park Ave., Suite S3, Minneapolis, MN 55415 USA; 2 Department of Family Medicine and Adult and Child Center for Outcomes Research and Delivery Science (ACCORDS), University of Colorado Anschutz Medical Campus, Aurora, CO USA; 3 Department of Family Social Science, University of Minnesota, St. Paul, MN USA; 4 Department of Family Medicine and Community Health, University of Minnesota Medical School, Minneapolis, MN USA

**Keywords:** Integrated behavioral health, Primary care, Statewide data sets, Systems change, Chronic diseases, Healthcare disparities, Health disparities, Health equity

## Abstract

**Background:**

People with diabetes, vascular disease, and asthma often struggle to maintain stability in their chronic health conditions, particularly those in rural areas, living in poverty, or racially or ethnically minoritized populations. These groups can experience inequities in healthcare, where one group of people has fewer or lower-quality resources than others. Integrating behavioral healthcare services into primary care holds promise in helping the primary care team better manage patients’ conditions, but it involves changing the way care is delivered in a clinic in multiple ways. Some clinics are more successful than others in fully integrating behavioral health models as shown by previous research conducted by our team identifying four patterns of implementation: Low, Structural, Partial, and Strong. Little is known about how this variation in integration may be related to chronic disease management and if IBH could be a strategy to reduce healthcare inequities. This study explores potential relationships between IBH implementation variation and chronic disease management in the context of healthcare inequities.

**Methods:**

Building on a previously published latent class analysis of 102 primary care clinics in Minnesota, we used multiple regression to establish relationships between IBH latent class and healthcare inequities in chronic disease management, and then structural equation modeling to examine how IBH latent class may moderate those healthcare inequities.

**Results:**

Contrary to our hypotheses, and demonstrating the complexity of the research question, clinics with better chronic disease management were more likely to be Low IBH rather than any other level of integration. Strong and Structural IBH clinics demonstrated better chronic disease management as race in the clinic’s location became more White.

**Conclusions:**

IBH may result in improved care, though it may not be sufficient to resolve healthcare inequities; it appears that IBH may be more effective when fewer social determinants of health are present. Clinics with Low IBH may not be motivated to engage in this practice change for chronic disease management and may need to be provided other reasons to do so. Larger systemic and policy changes are likely required that specifically target the mechanisms of healthcare inequities.

## Introduction

Generally in the U.S., non-Hispanic White and Asian people experience a superior quality of healthcare than Black, American Indian/Alaska Native, and Latinx people. [1]. Geography and socioeconomic status also seriously impact people’s ability to access quality healthcare [1]. These inequities are particularly potent for chronic diseases such as diabetes, vascular disease, and asthma, and have wide-ranging causes as well as implications for individual patients and their well-being [1]. Ensuring equity in the access and availability of high-quality care for all patients can and should be a goal of any healthcare provider or organization. In the present study, we examined how integrated behavioral health relates to chronic disease management inequities among a community sample of clinics.

## Chronic disease: health and healthcare inequities

There are many diseases that can be labeled chronic; for the purpose of this study, we focus on asthma, diabetes, and vascular disease. All three of these diseases have significant health inequities for non-White populations in the U.S. For example, in 2018 14.2% of non-Hispanic Black children had an asthma diagnosis, compared to 6.8% of non-Hispanic White children [1]. Not only do Black children experience asthma at over twice the rate of White children, they are over four times more likely to experience a hospitalization for asthma than White children, indicating inequities in both contributing factors and possible preventive healthcare quality and/or access discrepancies [1]. Cardiovascular disease in adults also is unequally distributed; the American Heart Association indicates that Blacks, Latinos, and Asians (including South Asians) experience both increased risk for cardiovascular disease as well as inequities in healthcare regarding preventive care and adverse cardiovascular events [2–4]. The American Heart Association also indicated in their reports that substantial variation exists within these large, diverse race/ethnicity subgroups (e.g., “Asian Americans” encompass those of Indian, Japanese, Thai, Chinese, Filipino, and other descents; a wide swath of the globe), and that little research has sufficiently disaggregated data on these subgroups. This aggregation of diverse groups is an example of structural racism in health research and care that both perpetuates generalizations among heterogeneous groups and also prevents better care from occurring [5].

Overlapping with racial/ethnic health inequities, there are significant inequities in the U.S. regarding access and quality of care along the spectrum of socioeconomic status. People with high income (above 400% of the federal poverty level) typically received higher quality care than those with lesser incomes on over half of the measures tracked by federal government agencies in 2016-2018 [1]. Healthcare quality was also worse for people who struggled with healthcare access due to finances or who had chronic medical conditions such as asthma or diabetes [1]. For example, people with public insurance reported about twice as often that providers didn’t respect what they had to say, compared to people with private insurance [1]. People without insurance were significantly less likely to have had their blood pressure checked in the last two years and children without insurance were significantly less likely to have had their height and weight checked in the last two years, compared to those with private insurance [1]. Both measures can be considered preventive care for the chronic diseases examined in this paper (e.g., diabetes, vascular disease).

Finally, location can be a contributing factor to worse health status or healthcare quality. Federal government surveys showed that nonmetropolitan (i.e., rural) areas performed worse on about a third of healthcare quality measures compared to suburban areas (which performed better than rural and urban). Particularly salient examples of areas where suburban areas demonstrated stronger metrics than rural areas included the percent of people who had received a cholesterol screening in the last 5 years (rural = 81.3%, suburban = 89%), and percent reporting that providers did not show respect for what patients had to say or did not listen carefully to them (rural = 8.9%, suburban = 5.3%) [1]. In addition, current inequities do not appear to have improved much since the 2002 measure [1].

## Integrated behavioral healthcare and inequities

A question remains about whether integrated behavioral healthcare (IBH) may be one mechanism by which healthcare inequities can be addressed. Integrated behavioral healthcare is a primary care practice model wherein behavioral or mental health professionals (e.g., licensed clinical social workers, licensed marriage and family therapists, psychologists, licensed professional counselors) are integrated into the primary care team and provide services such as follow-up on positive screenings, brief therapy, connections to specialty mental healthcare, health behavior change, and crisis management [6]. Generally, IBH has been found to increase access to behavioral healthcare and improves clinical outcomes for both chronic medical conditions and mental illness [7–14]. Some research demonstrates that IBH can result in improved access to mental healthcare for individuals and families with risk factors such as poverty, [15–17]. rurality, [18–20] and being members of diverse racial/ethnic minorities or limited-English populations, [21–23] when targeted approaches are used for these populations. IBH is part of a “whole person” approach to healthcare, and therefore can result in better adherence to treatment plans due to increasing patient engagement, eliciting patient concerns and activating the patient with problem-solving barriers to health [24] What has not been examined in previous research is whether IBH can improve access to behavioral healthcare on a clinic or population level and if so, how well-implemented IBH needs to be in order to make that impact, or what specific elements of IBH are most important in making that impact. We still need to learn whether, given the level of IBH implementation fidelity achieved by a sample of community clinics, there is a difference in healthcare management outcomes for these groups subject to inequities.

## Use of previously established latent classes of IBH in clinics

**Fig. 1:**
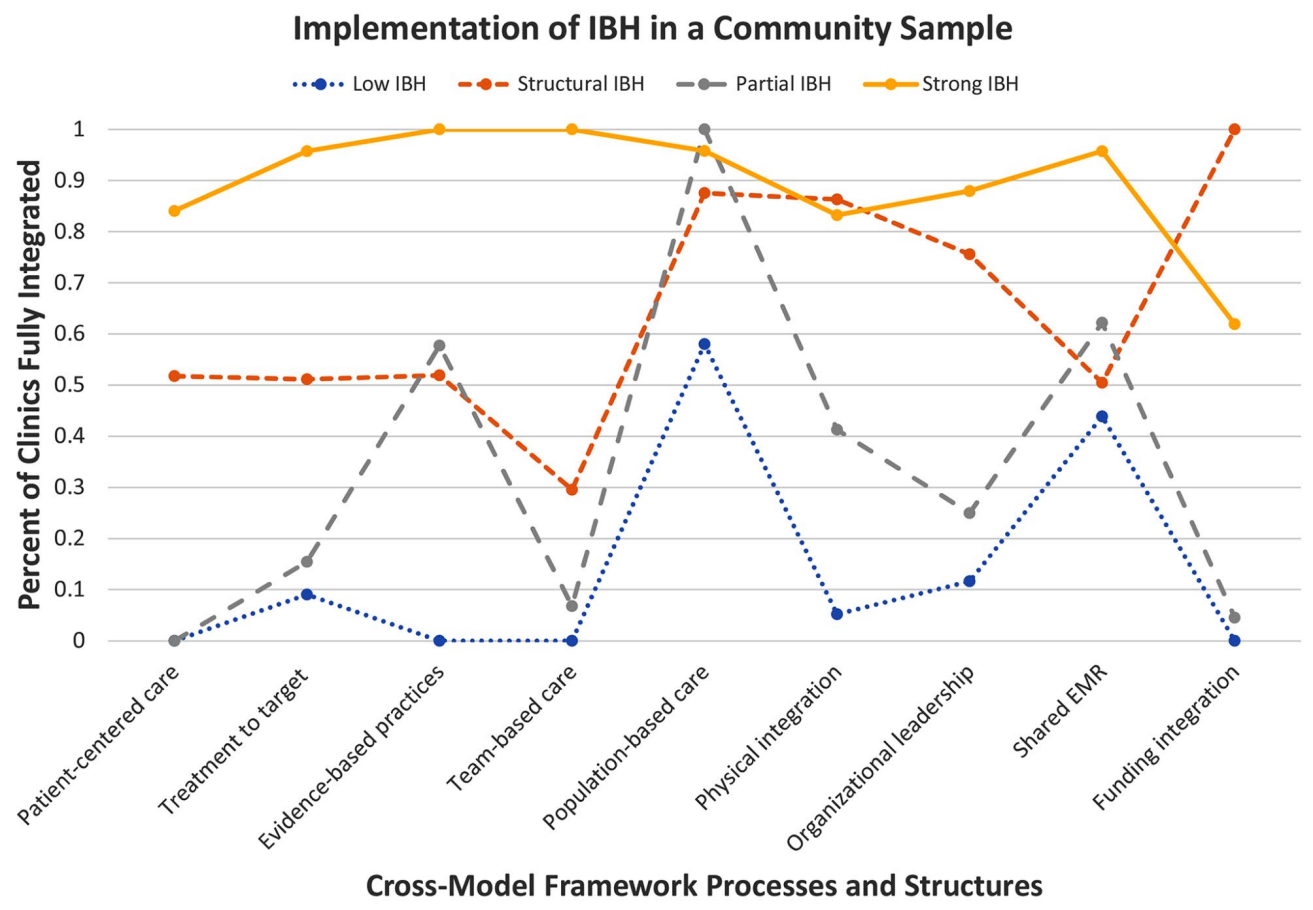
Four-class latent class solution. (From Buchanan GJR, Piehler T, Berge J, Hansen A, Stephens KA. Integrated behavioral health implementation patterns in primary care using the cross-model framework: A latent class analysis. Adm Policy Ment Heal Ment Heal Serv Res. 2021;(0123456789). doi:10.1007/s10488-021-01165-z)

IBH is not a monolithic construct, but involves multiple elements that all may be implemented with variability across clinics. Due to this potential for variability by element, the present study utilizes a latent class analysis previously published [25] by the authors (see Fig. 1) based on Stephens’ IBH Cross-Model Framework, [26] which specifies the five principles (groups of clinic processes and characteristics of the care team) and nine structures (clinic structures needed to support IBH) critical to IBH. The Cross-Model Framework intentionally is model-agnostic, meaning it can be used to assess Collaborative Care programs as well as Primary Care Behavioral Health and co-location [26] A latent class analysis is a person-centered (or in this case, a clinic-centered) type of analysis used when there appears to be some heterogeneity in a sample on a set of indicators [27] This statistical approach can find unobserved groups within the sample that are similar to each other on a set of variables, to the point where they can be considered a “class.” In our latent class analysis, we layered the Cross-Model Framework over a previously developed questionnaire, the Site Self-Assessment. We were able to match the five principles: (1) patient-centric care, (2) treatment to target, (3) use evidence-based behavioral treatments, (4) conduct efficient team care, and (5) population-based care; and four of the structures: (6) sustainable fiscal strategies, (7) physical integration, (8) organizational leadership support for integrated care, and (9) shared EHR system. We used these nine components of the Cross-Model Framework as our indicator variables through which we identified the four IBH latent classes described next.

Our analysis found four classes of clinics demonstrating four different implementation patterns of the Cross-Model Framework, including Low IBH (39.6% of the sample), Structural IBH (7.9%), Partial IBH 29.4%), and Strong IBH (23.1%) [25] Low IBH clinics rarely met criteria for any of the nine examined Cross-Model Framework components. Structural IBH clinics typically met criteria on population-based care, physical integration, organizational leadership, and funding integration. Partial IBH clinics typically met criteria for population-based care and shared EMR. Strong IBH clinics met criteria for all nine components except funding integration. This study demonstrated that IBH implementation is not as simple as “yes, the clinic has IBH” or “no, the clinic does not have IBH.” Rather, different types of clinics tend to implement key pieces of the IBH approach to patient care differently, even excluding some aspects of IBH altogether. The four newly-discovered patterns provide an opportunity to examine healthcare inequities through a more nuanced lens than has been done previously. Previous research [28, 29] has indicated that clinics “with IBH” tend to provide better care for minoritized populations and those with fewer economic resources. But no research that we are aware of has acknowledged in their study design or research questions that IBH may be inconsistently implemented in IBH clinics and whether certain components of IBH might be more essential than others. For example, there may be elements of the IBH model that are more essential than others in seeing improved outcomes for minoritized and low-resource patients.

### The current study

The current study aimed to address the gap in research regarding how variability in IBH implementation relates to differences in healthcare inequities. Among a community sample of clinics, the present study specifically aims to: (1) determine the direct relationship between IBH implementation latent classes and chronic disease management outcomes, (2) determine the direct relationship between clinic-level context variables including rurality, patient socioeconomic risk, and patient race/ethnicity, and chronic disease management outcomes, otherwise known as healthcare inequities, and (3) determine the moderating effects of IBH implementation variation, in the form of latent classes, on the revealed healthcare inequities. Based on the demonstrated gap in research for chronic disease management, the focus was the success of healthcare management in achieving stability within accepted clinical ranges of outcomes for patients.

## Methods

### Participants

Family medicine, pediatrics, and internal medicine clinics (*n* = 102) across 14 healthcare organizations in the state of Minnesota completed questionnaires. Clinics were located across the state, including urban, suburban, and rural settings, and served approximately 1.5 million patients, or about 27% of the population of Minnesota. They were all part of healthcare systems which participated in the Institute for Clinical Systems Improvement’s (ICSI) integrated behavioral healthcare working group. ICSI is a regional non-profit focused on building collaborations among healthcare systems for quality improvement. Participants and procedures are more fully described by Buchanan et al [25] See Table 1 for clinic descriptives.

### Procedures

This study includes three primary sources of information: (1) Site Self-Assessment surveys [30] measuring IBH completed by the 102 clinics in 2018–2019 through the Institute of Clinical Systems Improvement, [25] (2) data for clinic SES risk scores and clinical outcome variables are from Minnesota Community Measurement (MNCM), the contracted data collection organization of the Minnesota Department of Health’s statewide healthcare quality reporting system, and (3) data collected from various secondary sources (detailed below). MNCM data were obtained in consultation with personnel at MNCM, who explained and recommended the use of the MNCM-determined risk scores as one way to examine healthcare inequities. See the MNCM Methodology report for 2018 for full explanation of data collection procedures [31] Data sources for clinic rurality and clinic area race/ethnicity make-up were publicly-accessible databases and are detailed below. The University of Minnesota IRB considered this study not human subjects research and therefore exempt from review.

### Measures

#### Clinic rurality

Rurality is based on the USDA Rural-Urban Commuting Area Codes (RUCA) of the clinic ZIP code (obtained from https://ruralhealth.und.edu/ruca*).* This scale ranges from 1 to 10 based on population density and commuting patterns, with 1 being urban and 10 being extremely remote.

#### Clinic-level patient risk

Determined by MNCM, this variable is a composite, clinic-level score of patient-level risk factors (i.e., health insurance product type (commercial, Medicare, Medicaid, uninsured, unknown), patient age, and deprivation index). Patient age was included because MNCM has determined that older patients are more compliant with treatment (G. Nelson, personal communication). The deprivation index is reflective of analysis of each clinic’s patient home address data. It includes patient ZIP code level averages of poverty, public assistance, unemployment, single female with child(ren), and food stamp usage. Each clinic has a unique risk score for each clinical outcome because the collective group of patients with each diagnosis was different.

#### Clinic area race/ethnicity make-up

Race/ethnicity for each clinic’s city location (incorporating the full city population) was obtained from the 2017 American Community Survey (obtained from https://www.census.gov/acs/www/data/data-tables-and-tools/data-profiles/2017/*).* Estimated counts and percentages of white, black, American Indian, Asian, Hawaiian/Pacific Islander, other, and two races/ethnicities were included.

#### Chronic disease management

Outcomes were obtained from publicly available data from MNCM, which obtains their data through a state government mandate to all healthcare organizations and clinics in the state [32] The following measures are required to be reported by all relevant clinics in the state through MNCM. Selected results are made publicly available. This latent variable was estimated from four indicator variables: adult optimal asthma control, child optimal asthma control, optimal diabetes control, and optimal vascular disease control. We chose to use a latent variable to encompass these outcomes due to high inter-correlations among the variables.

#### Adult and child optimal asthma control

Optimal asthma control is defined as a patient achieving the following: “(1) Asthma well-controlled as defined by the most recent asthma control tool result and (2) Patient not at risk of exacerbation (i.e., fewer than two emergency department visits and/or hospitalizations due to asthma in the last 12 months).” [33] Adults included were ages 18–50 and children included were ages 5–17.

#### Adult optimal vascular care

Optimal vascular care is defined as a patient ages 18–75 with ischemic vascular disease achieving all four of the following: “(1) blood pressure less than 140/90 mmHg, (2) on a statin medication, unless allowed contraindications or exceptions are present, (3) non-tobacco use, and (4) on daily aspirin or anti-platelets, unless allowed contraindications or exceptions are present.” [33].

#### Adult optimal diabetes care

Optimal diabetes care is defined as a patient ages 18–75 with Type I or Type II diabetes achieving all five of the following: “(1) HbA1c less than 8.0 mg/dL, (2) blood pressure less than 140/90 mmHg, (3) on a statin medication, unless allowed contraindications or exceptions are present, (4) non-tobacco use, (5) patient with ischemic vascular disease on daily aspirin or anti-platelets, unless allowed contraindications or exceptions are present.” [33].

### Data analysis

The goal of the analysis was to evaluate the relationship between IBH implementation and healthcare inequities through three analyses. First, we used structural equation models to examine the direct relationship between IBH latent class assignment and chronic disease management (Aim 1) and then the direct relationship between the clinic context variables and chronic disease management (to assess for healthcare inequities; Aim 2). Third, we used a mixture model to examine the moderating effect of IBH latent classes on the relationship between the clinic context variables and the three separate outcomes. We utilized the BCH manual 3-step approach in Mplus 8.3 to first model the latent classes and then to examine their relationship with the distal outcome [34–36] This rigorous approach incorporates classification uncertainty when evaluating class-related outcomes and has been shown to outperform other related methodologies in managing bias [37] After completing the model, we assessed both the within-class relationships between clinic context variables and chronic disease management, as well as between-class differences in those relationships. Because we made no specific hypotheses of which classes might vary and in what manner, we examined all pairwise class differences to assess the totality of the differences among clinic context, chronic disease management, and IBH latent class.

To account for data non-independence in this multilevel dataset, we utilized the COMPLEX feature in Mplus 8.4 to adjust the standard errors. To manage missing data (14.7–33.3%), Mplus 8.4 implements full information maximum likelihood (FIML) [38] Missing data appeared to primarily occur where a service was irrelevant, for example, clinics that only served adults did not report child asthma management rates. There was also missing data on clinic size due to the survey participants not reporting it. Due to the large number of tests run, we accounted for the potential for Type II errors (i.e., false positives) through utilization of the Benjamini-Hochberg procedure (false discovery rate was set for 0.1 or 10% in this paper) [39] Possible false discoveries are indicated in the results tables; these findings were not examined further.

## Results

### Descriptives and bivariate correlations

The four variables that were used as indicators of the latent construct variable of chronic disease management (optimal control rates for adult asthma, child asthma, diabetes, and vascular disease) averaged from 44.8% for optimal diabetes management to 58.1% for vascular disease. See Table 1 for full descriptives and correlations. Correlations between clinic context variables and chronic disease management were generally strong. More rural clinics had poorer child asthma control. Clinics serving lower SES patients had poorer management of all four chronic diseases. Clinics serving more White racial/ethnic areas had significantly better adult asthma, diabetes and vascular disease management.

**Table 1:** Descriptives and correlations in study variables

Variable	M	SD	*N*	Clinic Rurality	Clinic SES risk	Clinic Area Race/ ethnicity (% White)	Adult Asthma	Child Asthma	Diabetes
Clinic Rurality^a^	2.2	2.6	102	-					
Clinic SES risk^b^	1.0	0.1	87	-0.15	-				
Clinic Area Race/ethnicity (% White)	79.7	13.3	99	0.46^**^	− 0.59^**^	-			
Adult Asthma (%)	49.6	17.4	86	-0.11	− 0.38^**^	0.25^*^	-		
Child Asthma (%)	58.0	0.2	68	− 0.31^*^	− 0.35^**^	0.04	0.87^**^	-	
Diabetes (%)	44.8	10.1	78	0.13	− 0.67^**^	0.49^**^	0.63^**^	0.53^**^	-
Vascular (%)	58.1	0.1	77	0.03	− 0.68^**^	0.35^**^	0.66^**^	0.55^**^	0.83^**^

Notes: *M* = mean, *SD* = standard deviation, *N* = number of clinics reporting

^a^ Clinic Rurality ranges 1–10 with 1 being most urban and 10 being most rural;

^b^ Clinic SES risk is a transformed variable where 1.0 indicates average risk, below 1.0 indicates lower-than-average risk and above 1.0 indicates higher-than-average risk

### Direct relationship between IBH latent class and chronic disease management

We examined differences between IBH classes in chronic disease management. There were two relative differences between classes: Low IBH clinics had significantly better chronic disease management than both Structural IBH clinics (*ΔM* = 12.5, *p* = .03) and Strong IBH clinics (*ΔM* = 4.81, *p* = .02). All results are displayed in Table 2.

**Table 2:** Chronic disease management outcomes descriptives and comparison by class

Latent Class	M^*^	SD	Compared with	ΔM^**^	SE	*p*
Low IBH (Class 1)	2.93	9.18	Structural IBH	**12.50**	**5.87**	**0.03**
			Partial IBH	0.43	3.49	0.90
			Strong IBH	**4.81**	**2.04**	**0.02**
Structural IBH (Class 2)	-9.57	3.63	Partial IBH	-12.07	6.86	0.08
			Strong IBH	-7.69	6.01	0.20
Partial IBH (Class 3)	2.50	11.38	Strong IBH	4.38	3.64	0.23
Strong IBH (Class 4)	-1.88	12.17				

Note: Means and standard errors are shown by class for the latent variable “chronic disease management” which was indicated by four chronic disease variables. The chronic disease management variable is mean-centered with class-specific means and SD noted in the table

^*^Mean-centered variable with higher scores indicating better management; ^**^The difference between the class means examined for significance through Wald chi-square tests

### Direct relationship between clinic context variables and chronic disease management (healthcare inequities)

Rurality and socioeconomic risk demonstrated the most robust healthcare inequities. For chronic disease management, as both rurality (*B* = -0.08, *p* = .01) and socioeconomic risk (*B* = -9.62, *p* < .001) increased, adequate management decreased. There was no significant association between race/ethnicity or organization size and chronic disease management. All results are displayed in Table 3.

**Table 3:** Results of structural equation models for clinic context variables regressed onto chronic disease management (healthcare inequities)

Clinic Context Variables	B	SE	*p*
SES risk	-9.62	2.09	**0.000**
Rurality	-0.08	0.03	**0.01**
Race/ethnicity	-0.18	0.14	0.18
Organization size	0.13	0.13	0.31
Clinic size (Active patient population)	0.08	0.09	0.40

### Relationship between IBH latent class and the clinic context-outcomes link: moderation and within-class effects

Separate analyses were run using each clinic context variable as a predictor variable with IBH latent classes as categorical moderating variables. First, for each analysis, we examined within-class relationships of clinic context and chronic disease management, then examined whether there were any significant differences in those relationships across classes. Rurality analysis included clinic area race/ethnicity (percent White) as a control variable and vice versa. We were unable to include any control variables in the socioeconomic risk analysis due to model non-convergence. All results are displayed in Table 4.

**Table 4:** Results of IBH class moderating the association between contextual variables and chronic disease management outcomes

Context Variable / Latent Class	B^*^	SE	*p*	Compared with	ΔB^**^	SE	*p*
**SES Risk ->** *Chronic Disease Management Outcomes*							
Low IBH (Class 1)	**-5.19**	**1.85**	**0.01**	Structural IBH	3.13	3.20	0.33
				Partial IBH	15.23	12.01	0.21
				Strong IBH	**4.97**	**1.47**	**0.001**
Structural IBH (Class 2)	**-8.32**	**3.43**	**0.02**	Partial IBH	12.10	13.32	0.36
				Strong IBH	1.85	3.55	0.60
Partial IBH (Class 3)	-20.42	11.35	0.07	Strong IBH	-10.26	11.87	0.39
Strong IBH (Class 4)	**-10.16**	**2.81**	**0.00**				
**Rurality ->** *Chronic Disease Management Outcomes*							
Low IBH (Class 1)	-0.06	0.18	0.72	Structural IBH	-0.74	0.61	0.23
				Partial IBH	11.25	89.61	0.90
				Strong IBH	0.09	0.28	0.74
Structural IBH (Class 2)	0.68	0.46	0.14	Partial IBH	11.99	89.67	0.89
				Strong IBH	0.83	0.79	0.29
Partial IBH (Class 3)	-11.31	89.63	0.90	Strong IBH	-11.15	89.48	0.90
Strong IBH (Class 4)	-0.16	0.40	0.69				
**Race/Ethnicity ->** *Chronic Disease Management Outcomes*							
Low IBH (Class 1)	0.34	0.32	0.29	Structural IBH	-0.46	0.29	0.12
				Partial IBH	0.06	0.43	0.88
				Strong IBH	-0.51	0.36	0.15
Structural IBH (Class 2)	**0.80**	**0.31**	**0.01**	Partial IBH	0.53	0.55	0.34
				Strong IBH	-0.05	0.53	0.93
Partial IBH (Class 3)	0.28	0.34	0.42	Strong IBH	**-0.58**	**0.28**	**0.04+**
Strong IBH (Class 4)	**0.85**	**0.31**	**0.01**				

Note:^+^ Using the Benjamini-Hochberg false discovery rate procedure, this test is no longer significant (not interpreted)

Note: ^*^Mean-centered variable with higher scores indicating better management; ^**^Wald chi-square tests

#### Socioeconomic risk

All classes except Partial IBH (which trended similarly) had significantly negative relationships between socioeconomic risk and chronic disease management. IBH latent class moderated the relationship such that Low IBH clinics had a significantly less negative relationship than Strong IBH clinics (Wald test, *ΔB* = 4.97, *p* = .001). This indicates that Low IBH clinics with a large portion of low SES patients manage chronic disease better than clinics with Strong IBH with similar patient populations.

#### Rurality

Counter to the overall healthcare disparities result, there were no significant relationships within or between any of the IBH latent classes for rurality and chronic disease management.

#### Race/ethnicity

Both Structural IBH clinics (*B* = 0.80, *p* = .01) and Strong IBH clinics (*B* = 0.85, *p* = .01) demonstrated a significantly positive relationship between a clinic’s area population being increasingly White and better chronic disease management. This indicates that clinics in these categories that are in areas with more White residents do better on this outcome than clinics in these classes that are in more diverse areas.

## Discussion

IBH can be a powerful tool in managing population-level mental and physical health and improving access to mental healthcare. Yet this study has demonstrated that there is nuance in the relationship between IBH and optimal chronic disease management outcomes. The inequities in chronic disease management present in this sample of clinics included rurality and SES risk, consistent with previous research, [1, 40] although we surprisingly observed an overall lack of health outcome inequities for racial/ethnic minorities. This could be because race and ethnicity measured at the clinic area level is not specific enough.

An unexpected finding was that Low IBH clinics had better chronic disease management. There are several possible explanations for this, including: (1) Low IBH clinics may devote more resources to chronic disease management and less to mental health, and therefore have better outcomes; or (2) In our previous study, there was some indication that clinics with more low SES patients were more likely to have implemented some IBH [25] This is consistent with research by Tong and colleagues [41] who found that family physicians who worked collaboratively with behavioral health professionals were more likely to work at FQHCs, government health clinics, and academic health centers, all of which are more likely to serve minoritized and under-resourced populations. Additionally, clinics with “vulnerable populations” of 10–49% were 46% more likely and above 50%, were 141% more likely, than those with less than 10% of these patients to have behavioral health collaborators. Therefore, it is likely that clinics with some form of IBH have more complex patients with fewer resources, and therefore more challenges managing chronic diseases, compared to Low IBH clinics. Providers and staff at Low IBH clinics may have felt less pressure to make changes in their care models and/or have less time and resources to make these changes, resulting in less movement towards integration with behavioral health.

### Socioeconomic risk

The first analysis examined whether any IBH classes seemed to have a different pattern than expected for healthcare inequities based on SES risk (low SES). SES risk was a strong predictor of poorer chronic disease management in most classes and it appears that overall, IBH implementation class was not associated with variability in healthcare management outcomes when SES risk is high. In our previous research, [25] Partial clinics (i.e., Structural IBH clinics typically met criteria on. Partial IBH clinics typically met criteria for population-based care and shared EMR.) also were shown to have a smaller proportion of patients with SES risk than Structural IBH (i.e., good population-based care, physical integration, organizational leadership, and funding integration) clinics so low SES patients may have been less represented in the Partial class. Therefore, there is not one pattern of IBH implementation for which there is a clear amelioration of SES risk.

### Rurality

In our previous research, [25] we found that Partial clinics were more likely to be urban than the other three types of clinics, and this may explain some of the insignificant results when it comes to the Partial clinics, given the healthcare inequities associated with rurality would not be present for those clinics. However, it is also likely that due to our study being underpowered generally, we did not detect any differences that may be present.

### Race/ethnicity

Regarding race/ethnicity, Structural and Strong IBH clinics had better chronic disease management outcomes as their area’s racial/ethnic diversity decreased. Low and Partial IBH clinics did not change in their chronic disease management across the diversity spectrum. The better chronic disease management in Strong and Structural IBH clinics found in Whiter areas is something to examine with more detail. This discrepancy doesn’t appear in the Low and Partial classes, which may mean that Strong or Structural IBH is helping improve chronic disease management for patients living in more White areas. It is known that social determinants of health (SDOH) and structural racism complicate successful management of chronic disease beyond what IBH is intended to address. It is also possible that IBH providers in more diverse areas may need to either (1) be diversified as a workforce or (2) receive better cultural competency training to better engage diverse patients and improve the other end of the spectrum [42]

### Social determinants of health

The results of this study emphasize the significant role that social determinants of health (SDOH) can play in the management of patients’ physical and mental health, even when a clinic has implemented IBH well. Indeed, research has demonstrated that medical care only accounts for approximately 10–20% of the population’s overall health, with social and economic factors far outweighing medical care [43] Our study demonstrates what IBH is and what it is not. Specifically, while IBH can be effective at increasing access to mental healthcare and even improving the quality of both mental and physical healthcare provided, it may only be part of a larger social care package – whether that is implemented inside or outside the healthcare organization – that is necessary to resolve healthcare inequities. IBH cannot resolve that problem on its own.

This paper also demonstrates that health outcomes can vary within the same IBH classes depending on context, such that Strong IBH may be associated with better healthcare management outcomes in Whiter areas but not necessarily in low SES areas. This could be related to what integrated behavioral health professionals are doing in these clinics. It is not enough to say a clinic “has integrated behavioral health,” but it must be measured, or at least described in the context of a well-known framework, so that it is clear what aspects of IBH are present and which are not, in order to better understand the relationship between IBH as it is implemented and the outcomes being reported.

### Strengths and limitations

Limitations of this study include first, its cross-sectional nature; there is no ability to state whether a clinic’s IBH implementation class causes or is caused by differences in the context of the clinics or in the patients they serve. Second, this is a snapshot in time, and we were not able to see how implementation and chronic disease management influenced each other over time. Third, due to the sample size and the complex nature of the analyses, robust control variables were not able to be used, and the high correlations between the predictor variables make it difficult to parse the variance. Fourth, the clinic racial and ethnic make-up were approximated from the clinic’s city rather than from the clinics themselves, and we did not find health inequities related to this variable, which indicates that the variable may have lacked adequate variability. Examining racial and ethnic make-up at the clinic level loses a significant amount of the variance seen at the individual level. Finally, self-report by the clinics regarding their IBH implementation status may have introduced measurement error due to self-report biases.

The present study was the first to examine chronic disease management and its inequities in the context of IBH implementation variation and uses a sample of more than 100 clinics that are in various stages of implementing IBH. Its strength lies in the real-world setting of clinics across an entire state and many different healthcare organizations which showed a large spread of IBH implementation, from none to full fidelity.

This study allows a more nuanced understanding of how various patterns of IBH components’ existence in clinics may relate to mitigating or exacerbating healthcare inequities. As a primarily hypothesis-generating study, this study met its goal of providing key research questions for future research. Future questions include understanding the implementation process for each principle and structure of the Cross-Model Framework, barriers to implementing specific principles and structures, and strategies for success. More should be learned about the experiences of providers and staff at clinics that have not implemented any IBH efforts and whether they feel the need to do so. Future research should also examine patient-level outcomes of chronic disease management in the context of the different classes of IBH.

## Data Availability

The data that support the findings of this study are available from Minnesota Community Measurement and the Institute for Clinical Systems Improvement, but restrictions apply to the availability of these data, which were used under a data use agreement for the current study and so are not publicly available. The data are, however, available from the authors upon reasonable request and with the permission of the above organizations. Additionally, some data utilized are publicly available from the 2017 American Community Survey (obtained from https://www.census.gov/acs/www/data/data-tables-and-tools/data-profiles/2017/).

## Abbreviations

IBH: Integrated behavioral health
ICSI: Institute for Clinical Systems Improvement
SES: Socioeconomic risk
SDOH: Social determinants of health

## References

[1] Agency for Healthcare Research and Quality. 2019 National Healthcare Quality and Disparities Report. 2020. https://www.ahrq.gov/sdoh/about.html.

[2] Volgman AS, Palaniappan LS, Aggarwal NT et al. *Atherosclerotic Cardiovascular Disease in South Asians in the United States: Epidemiology, Risk Factors, and Treatments: A Scientific Statement From the American Heart Association*. Vol 138.; 2018. 10.1161/CIR.0000000000000580. 10.1161/CIR.0000000000000580 29794080

[3] Rodriguez CJ, Allison M, Daviglus ML, et al. Status of Cardiovascular Disease and Stroke in Hispanics/Latinos in the United States: A Science Advisory from the American Heart Association. 130. 2014. 10.1161/CIR.0000000000000071. 10.1161/CIR.0000000000000071 PMC4577282 25098323

[4] Carnethon MR, Pu J, Howard G et al. *Cardiovascular Health in African Americans: A Scientific Statement From the American Heart Association*. Vol 136.; 2017. 10.1161/CIR.0000000000000534. 10.1161/CIR.0000000000000534 29061565

[5] Churchwell K, Elkind MSV, Benjamin RM, et al. Call to action: structural racism as a fundamental driver of health disparities: a presidential advisory from the American Heart Association. Circulation. 2020;142(24). 10.1161/CIR.0000000000000936. 10.1161/CIR.0000000000000936 33170755

[6] Peek CJ, The National Integration Academy Council. Lexicon for Behavioral Health and primary care integration: concepts and definitions developed by Expert Consensus. AHRQ publication No.13-IP001-EF. Agency for Healthcare Research and Quality; 2013. https://integrationacademy.ahrq.gov/sites/default/files/Lexicon.pdf.

[7] Pomerantz AS, Cole BH, Watts BV, Weeks WB. Improving efficiency and access to mental health care: combining integrated care and advanced access. Gen Hosp Psychiatry. 2008;30(6):546–51. 10.1016/j.genhosppsych.2008.09.004. 19061681 10.1016/j.genhosppsych.2008.09.004

[8] Vickers KS, Ridgeway JL, Hathaway JC, Egginton JS, Kaderlik AB, Katzelnick DJ. Integration of mental health resources in a primary care setting leads to increased provider satisfaction and patient access. Gen Hosp Psychiatry. 2013;35(5):461–7. 10.1016/j.genhosppsych.2013.06.011. 23910217 10.1016/j.genhosppsych.2013.06.011

[9] Sarvet B, Gold J, Bostic JQ, et al. Improving access to mental health care for children: the Massachusetts Child Psychiatry Access Project. Pediatrics. 2010;126(6):1191–200. 10.1542/peds.2009-1340. 21059722 10.1542/peds.2009-1340

[10] Campo JV, Geist R, Kolko DJ. Integration of Pediatric Behavioral Health Services in primary care: improving Access and outcomes with Collaborative Care. Can J Psychiatry. 2018;63(7):432–8. 10.1177/0706743717751668. 29673268 10.1177/0706743717751668 PMC6099777

[11] Dollar KM, Kearney LK, Pomerantz AS, Wray LO. Achieving same-day access in integrated primary care. Fam Syst Heal. 2018;36(1):32–44. 10.1037/fsh0000327. 10.1037/fsh0000327 29369648

[12] Pomerantz AS, Kearney LK, Wray LO, Post EP, McCarthy JF. Mental health services in the medical home in the Department of Veterans affairs: factors for successful integration. Psychol Serv. 2014;11(3):243–53. 10.1037/a0036638. 24841512 10.1037/a0036638

[13] Miller-Matero LR, Khan S, Thiem R, DeHondt T, Dubaybo H, Moore D. Integrated primary care: patient perceptions and the role of mental health stigma. Prim Heal Care Res Dev 2018;(June. 2015;1–4. 10.1017/S1463423618000403. 10.1017/S1463423618000403 PMC6567893 29914587

[14] Butler M, Kane RL, McAlpine D, et al. Integration of mental health/substance abuse and primary care 173 (prepared by the Minnesota evidence-based Practice Center under contract 290-02-0009). AHRQ Publ 09- E003. 2008;173:1–362. 19408966. PMC4781124 19408966

[15] Cole MB, Qin Q, Sheldrick RC, Morley DS, Bair-Merritt MH. The effects of integrating behavioral health into primary care for low‐income children. Health Serv Res. 2019;54(6):1203–13. 10.1111/1475-6773.13230. 31742687 10.1111/1475-6773.13230 PMC6863244

[16] Hodgkinson S, Godoy L, Beers LS, Lewin A. Improving mental health access for low-income children and families in the primary care setting. Pediatrics. 2017;139(1):e20151175. 10.1542/peds.2015-1175. 27965378 10.1542/peds.2015-1175 PMC5192088

[17] Ogbeide SA, Landoll RR, Nielsen MK, Kanzler KE. To go or not go: patient preference in seeking specialty mental health versus behavioral consultation within the primary care behavioral health consultation model. Fam Syst Heal. 2018;36(4):513–7. 10.1037/fsh0000374. 10.1037/fsh0000374 30307267

[18] Valleley RJ, Kosse S, Schemm A, Foster N, Polaha J, Evans JH. Integrated primary care for children in rural communities: an examination of patient attendance at collaborative behavioral health services. Fam Syst Heal. 2007;25(3):323–32. 10.1037/1091-7527.25.3.323. 10.1037/1091-7527.25.3.323

[19] Logan DE, Lavoie AM, Zwick WR, Kunz K, Bumgardner MA, Molina Y. Integrating addiction medicine into rural primary care: strategies and initial outcomes. J Consult Clin Psychol. 2019;87(10):952–61. 10.1037/ccp0000410. 31556671 10.1037/ccp0000410

[20] Burt JD, Garbacz SA, Kupzyk KA, Frerichs L, Gathje R. Examining the utility of behavioral health integration in well-child visits: implications for rural settings. Fam Syst Heal. 2014;32(1):20–30. 10.1037/a0035121. 10.1037/a0035121 24684153

[21] Sanchez K, Watt TT. Collaborative care for the treatment of depression in primary care with a low-income, Spanish-speaking population: outcomes from a community-based program evaluation. Prim Care Companion J Clin Psychiatry. 2012;14(6):1–15. 10.4088/PCC.12m01385. 10.4088/PCC.12m01385 PMC3622537 23585998

[22] Holden K, McGregor B, Thandi P, et al. Toward culturally centered integrative care for addressing mental health disparities among ethnic minorities. Psychol Serv. 2014;11(4):357–68. 10.1037/a0038122. 25383991 10.1037/a0038122 PMC4228792

[23] Bridges AJ, Andrews AR, Villalobos BT, Pastrana FA, Cavell TA, Gomez D. Does integrated behavioral health care reduce mental health disparities for latinos? Initial findings. J Lat Psychol. 2014;2(1):37–53. 10.1037/lat0000009. 25309845 10.1037/lat0000009 PMC4193502

[24] Twomey J, Steinberg J, Whole-Person, Care. Implementing Behavioral Health Integration in the Patient-Centered Medical Home Models of Integrated Care : Published online 2016.

[25] Buchanan GJR, Piehler T, Berge J, Hansen A, Stephens KA. Integrated behavioral health implementation patterns in primary care using the cross-model framework: a latent class analysis. Adm Policy Ment Heal Ment Heal Serv Res. 2021;012345678910.1007/s10488-021-01165-z. 10.1007/s10488-021-01165-z PMC8854330 34529202

[26] Stephens KA, Van Eeghen C, Mollis B, et al. Defining and measuring core processes and structures in integrated behavioral health in primary care: a cross-model framework. Transl Behav Med. 2020;10(3):527–38. 10.1093/tbm/ibz163. 32766871 10.1093/tbm/ibz163 PMC8128511

[27] Oberski D. Mixture models: Latent Profile and Latent Class Analysis. Published Online. 2016;275–87. 10.1007/978-3-319-26633-6_12.

[28] Satcher D, Rachel SA. Promoting mental health equity: the role of integrated care. J Clin Psychol Med Settings. 2017;24(3–4):182–6. 10.1007/s10880-016-9465-8. 27628200 10.1007/s10880-016-9465-8

[29] O’Loughlin K, Donovan EK, Radcliff Z, Ryan M, Rybarczyk B. Using integrated behavioral healthcare to address behavioral health disparities in underserved populations. Transl Issues Psychol Sci. 2019;5(4):374–89. 10.1037/tps0000213. 10.1037/tps0000213

[30] Scheirer MA, Leonard BA, Ronan L, Boober BH. *Site Self Assessment Tool for the Maine Health Access Foundation Integration Initiative*.; 2010.

[31] Minnesota Community Measurement. *Depression Care in Minnesota – 2018 Report Appendix Methodology*.; 2018.

[32] MN Community Measurement. Our History. Published 2020. Accessed October 1. 2021. https://mncm.org/about/#our-history.

[33] Minnesota Community Measurement. *Quality of Care for Chronic Conditions in 2018 Report*.; 2018.

[34] McLarnon MJW, O’Neill TA. Extensions of auxiliary variable approaches for the investigation of mediation, moderation, and conditional effects in mixture models. Organ Res Methods. 2018;21(4):955–82. 10.1177/1094428118770731. 10.1177/1094428118770731

[35] Asparouhov T, Muthén B. Auxiliary variables in mixture modeling: using the BCH method in Mplus to estimate a distal outcome model and an arbitrary second model. Mplus Web Notes. 2019;21:1–27.

[36] Nylund-Gibson K, Grimm RP, Masyn KE. Prediction from latent classes: a demonstration of different approaches to include distal outcomes in mixture models. Struct Equ Model. 2019;26(6):967–85. 10.1080/10705511.2019.1590146. 10.1080/10705511.2019.1590146

[37] Dziak JJ, Bray BC, Zhang J, Zhang M, Lanza ST. Comparing the performance of improved classify-analyze approaches for distal outcomes in latent profile analysis. Methodology. 2016;12(4):107–16. 10.1027/1614-2241/a000114. 28630602 10.1027/1614-2241/a000114 PMC5473653

[38] Muthén LK, Muthén BO. Mplus user’s guide. Eighth Edi. Muthén & Muthén; 2017. https://www.statmodel.com/.

[39] Benjamini Y, Hochberg Y. Controlling the false Discovery rate: a practical and powerful Approach to multiple testing. J R Stat Soc Ser B. 1995;57(1):289–300. 10.1111/j.2517-6161.1995.tb02031.x. 10.1111/j.2517-6161.1995.tb02031.x

[40] Powers DM, Bowen DJ, Arao RF, et al. Rural clinics implementing collaborative care for low-income patients can achieve comparable or better depression outcomes. Fam Syst Heal. 2020;38(3):242–54. 10.1037/fsh0000522. 10.1037/fsh0000522 32700931

[41] Tong ST, Morgan ZJ, Stephens KA, Bazemore A, Peterson LE. Characteristics of Family Physicians practicing collaboratively with behavioral Health professionals. Ann Fam Med. 2023;21(2):157–60. 10.1370/afm.2947. 36973057 10.1370/afm.2947 PMC10042557

[42] Legha RK, Miranda J. An Anti-racist Approach To Achieving Mental Health Equity in Clinical Care. Psychiatr Clin North Am. 2020;43(3):451–69. 10.1016/j.psc.2020.05.002. 32773074 10.1016/j.psc.2020.05.002

[43] Hood CM, Gennuso KP, Swain GR, Catlin BB. County Health rankings: relationships between determinant factors and Health outcomes. Am J Prev Med. 2016;50(2):129–35. 10.1016/j.amepre.2015.08.024. 26526164 10.1016/j.amepre.2015.08.024

